# Supplementary material for: Exploring the Chemical Constituents, Antioxidant, Xanthine Oxidase and COX Inhibitory Activity of Commiphora gileadensis Commonly Grown Wild in Saudi Arabia
Source: Molecules. 2023 Mar 2;28(5):2321. doi: 10.3390/molecules28052321 (PMC10004785; doi:10.3390/molecules28052321)
Supplement: Supplementary file 1 [file molecules-28-02321-s001.zip › molecules-2227691-supplementary.pdf]

Article

Exploring the Chemical Constituents, Antioxidant, Xanthine Oxidase and COX Inhibitory Activity of *Commiphora gileadensis* Commonly Grown Wild in Saudi Arabia

Khalid A. Shadid [1], Ashok K. Shakya [1,*], Rajashri R. Naik [2], Talal S. Al-Qaisi [2], Ghaleb A. Oriquat [2], Ali M. Atoom [2] and Husni S. Farah [2]

[1] Pharmacological and Diagnostic Research Center, Department of Pharmaceutical Sciences, Faculty of Pharmacy, Al-Ahliyya Amman University, Amman-19328, Jordan
[2] Pharmacological and Diagnostic Research Center, Faculty of Allied Medical Sciences, Al-Ahliyya Amman University, Amman-19328, Jordan
* Correspondence: ak_shakya@ammanu.edu.jo; Tel: +962-5-3500211 (ext. 2135)

Supplementary Material

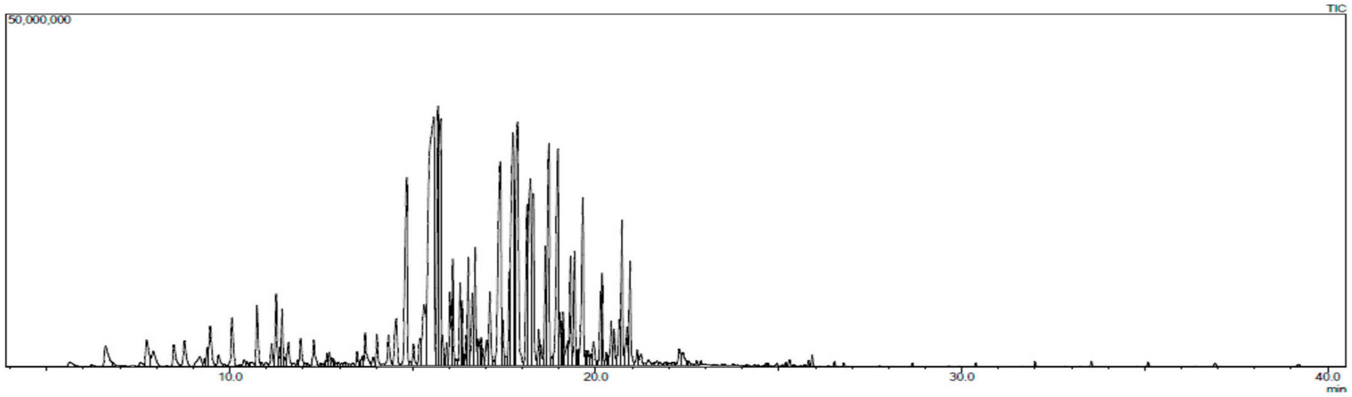

Figure S1. GC-MS analysis of ethanolic extract of aerial parts of *Commiphora gileadensis*.

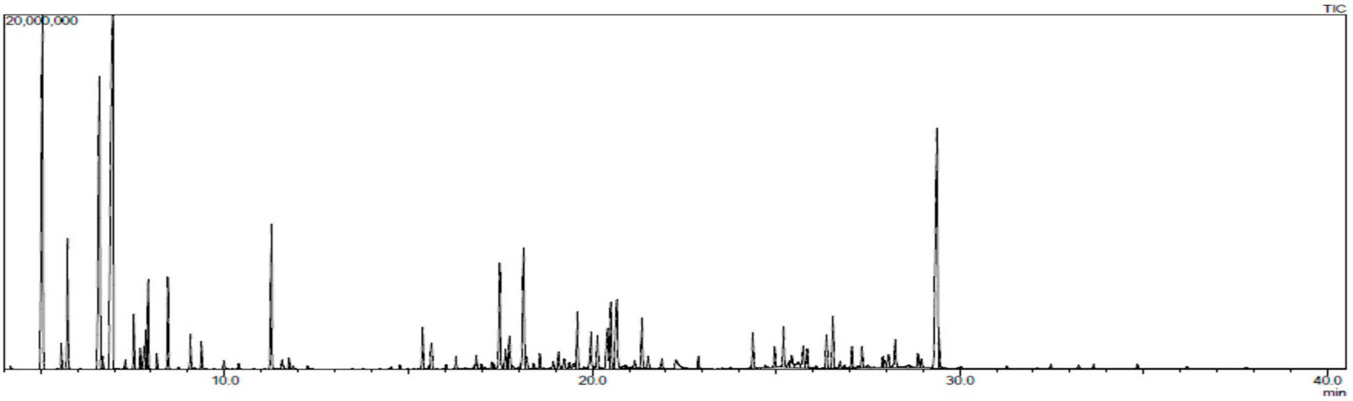

Figure S2. GC-MS analysis of Essential oil collected from aerial parts of *Commiphora gileadensis*.
